# Editorial: Unraveling the molecular web of inflammation and fibrosis: pathways, immune interactions, epigenetics, and therapeutic frontiers

**DOI:** 10.3389/fimmu.2026.2027878

**Published:** 2026-10-28

**Authors:** Hector A. Cabrera-Fuentes, Guillermo Barreto, William A. Boisvert

**Affiliations:** 1 División de Estudios de Posgrado e Investigación, Tecnológico Nacional de México/Instituto Tecnológico de Tijuana, Tijuana, Mexico; 2 UNAM-UABJO Research Center, Faculty of Medicine and Surgery, Universidad Autónoma Benito Juárez de Oaxaca, Oaxaca, Mexico; 3 Hospital General de Zona No.1 Dr. Demetrio Mayoral Pardo, Instituto Mexicano del Seguro Social (IMSS), Oaxaca, Mexico; 4 Université de Lorraine, CNRS, Laboratoire IMoPA, UMR 7365, Nancy, France; 5 Center for Cardiovascular Research, John A. Burns School of Medicine, University of Hawaii at Manoa, Honolulu, HI, United States

**Keywords:** epigenetic regulation, fibroblast plasticity, fibrosis, immunometabolism, inflammation, macrophage polarization, therapeutic targeting

Fibrosis is a prominent feature of most chronic inflammatory diseases (1) and is estimated to contribute to as much as 45% of deaths in industrialized countries (2). The fibrotic processes of lung, liver, kidney, heart and skin are initiated by different injuries yet converge in their later stages on a restricted set of signalling responses, among them transforming growth factor beta (TGF-β), platelet-derived growth factor and WNT (3). Where inflammation fits in that sequence has been harder to define. While failure of immunosuppression to alter the course of idiopathic pulmonary fibrosis moved inflammation away from the center of the field, lineage tracing of the alveolar fibroblast has placed an inflammatory fibroblast state upstream of the matrix-producing one, showing that preventing the fibrotic transition abrogates fibrosis while leaving inflammation worse (4). Fibroblasts across tissues resolve into conserved transcriptional subtypes that act as a reservoir for the activated states seen in certain diseases (5), and their plasticity is now treated as a therapeutic variable rather than a descriptive one (6, 7).

This Research Topic is represented by 20 contributions: nine original research articles, nine reviews and two mini reviews, covering lung, liver, kidney, heart, breast, skin, eye, joint, peritoneum, pelvic floor, airway, systemic vasculature and the oral cavity. We have grouped them by the regulatory mechanism(s) probed by each: fibroblast state transitions, the immune partners that set those states, epigenetic and post-transcriptional control, metabolic reprogramming, cross-organ convergence with its candidate biomarkers, and therapeutic strategy.

## Contribution of fibroblasts to local inflammation

Two contributions put the fibroblast transition itself at the centre of the problem. Panagiotidis et al. propose a reversible lipofibroblast to inflammatory lipofibroblast to activated myofibroblast switch in the alveolus, with IL-1β and IL-17A sustaining the inflammatory state and TGF-β driving the conversion to Cthrc1+ Acta2+ myofibroblasts, and with reversion to a lipogenic phenotype accompanying resolution. Their interpretation of the primary lineage-tracing data is more cautious: the frequency of inflammatory fibroblasts changes little during progression, and these cells lie adjacent to fibroblastic foci rather than within them. This observation is consistent with these cells functioning as a persistent partner population rather than necessarily as an obligatory intermediate. This ambiguity is one of the prominent open questions in this collection, because an intermediate can be bypassed whereas a neighbour must be engaged separately. Bhatt et al. approach the same transition through PI3K/Akt, which couples apoptosis resistance and senescence to fibroblast activation and epithelial-mesenchymal transition (EMT), and which sustains a senescence-associated secretory phenotype in alveolar epithelial cells. They note that the pathway is activated in idiopathic pulmonary fibrosis without PI3K mutations, so the target is signalling tone rather than genotype, and they propose phosphorylated Akt in bronchoalveolar lavage macrophages as a pharmacodynamic readout already shown to be measurable in patients.

## Immune cells that influence fibroblast phenotype

Three reviews and one model study specify which immune populations shape a fibroblast phenotype. Sun et al. show that the T cell contribution to lung fibrosis is multifactorial in that Th1-derived interferon gamma restrains fibrosis, Th2 interleukins 4 and 13 and Th9-derived IL-9 promote fibroblast activation and matrix deposition, IL-17A acts through IL-17RA, and that the net effect of regulatory T cells depends ultimately on disease stage. Their most useful mechanistic thread is the PD-1 positive Th17 population that drives collagen deposition through STAT3-dependent IL-17A and TGF-β1. Cai et al. describe the reciprocal circuit across multiple tissues: fibroblasts release CXCL1, CXCL2, CXCL8 and CXCL12 to recruit neutrophils through CXCR2 and CXCR4, and neutrophils return neutrophil extracellular traps, elastase and cytokines that change fibroblast behaviour. They also highlight a potential safety signal: 21 reported cases of inflammatory bowel disease were described in association with secukinumab or ixekizumab exposure. These reports reinforce the need to consider the tissue-specific protective functions of IL-17, including intestinal host defence, when evaluating pathway inhibition. Zheng et al. then demonstrate that the inflammatory program is not fixed across the lifespan. They describe in neonatal mice subjected to aortocaval fistula at postnatal day 7, pulmonary small vessel remodelling at day 30 was accompanied by 304 differentially expressed genes dominated by type I interferon and antiviral response terms, not by the IL-1β, IL-6 and tumour necrosis factor alpha profile of adult pulmonary hypertension models. Cyclosporin A and the type I interferon receptor blocker MAR1-5A3 each reduced remodelling, and the authors are appropriately careful to call the signature antiviral-like rather than viral.

## Epigenetic and post-transcriptional control converging on NF-κB

Four original research articles reach similar transcriptional output through four different RNA-level mechanisms. Ding et al. identify YTHDC1 as the m6A reader that destabilises the anti-inflammatory long non-coding RNA *LINC01579* in ankylosing spondylitis. They show that Xinfeng capsule lowers YTHDC1, restores *LINC01579* and suppresses IL-17/NF-κB signalling in patient-derived synoviocytes and in proteoglycan-induced arthritis, whereas the IL-17 agonist SR0987 reverses the benefit. Liu et al., working in cecal ligation and puncture peritonitis, place IKZF1 upstream of histone acetylation wherein IKZF1 recruits HDAC3 to the *Sdhb* promoter, H3K9 deacetylation and *SDHB* fall, succinate accumulates, HIF-1α is stabilised, and lenalidomide or acetate supplementation reverses each step while reducing both peritoneal inflammation and secondary pulmonary fibrosis by Ashcroft score. Liao et al. show that a circular RNA derived from the CTNNA1 gene (hsa_circ_0074158; its mouse homolog is termed circ_Ctnna1) binds EIF4A3 and destabilises its own host transcript, lowering alpha-catenin and opening the endothelial barrier in sepsis. Liu et al. report similar results with the opposite sign in severe asthma, where loss of *circ-0001875* releases *miR-31-5p*, reduces SP1 and permits M1 macrophage polarisation through NF-κB, in a cohort of 34 severe, 32 mild-to-moderate and 35 control subjects. Taken together, these four papers suggest that distinct regulatory mechanisms can converge on overlapping inflammatory outputs; their regulatory layers should not be considered interchangeable.

## Metabolism as an effector arm

Metabolic rewiring appears in this collection as a mechanism rather than a correlate, consistent with its treatment as a druggable axis in fibrosis (8). Feng et al. report that activating transcription factor 4 (ATF4) is upregulated in the obstructed kidney, predominantly in tubular epithelium. ATF4 knockdown reduces CCL2, tumour necrosis factor alpha, IL-1β, alpha-smooth muscle actin and fibronectin; the study further identifies the hexokinase II promoter as an ATF4-associated regulatory target, and pharmacological inhibition of glycolysis with 2-deoxy-D-glucose reproduces the antifibrotic effect. Similarly, succinate accumulation occurs downstream of *SDHB* repression in the peritonitis study above, and TGF-β-driven glycolysis with hexokinase occurs as a node in the alveolar review. Yao et al. add the oxidative arm, discussing how ferroptotic death of alveolar epithelial cells may release damage-associated molecular patterns that engage TLR4 and RAGE. They also review evidence suggesting that oxidised phospholipid signalling may influence macrophage phenotype through PPARγ, while matrix stiffening can activate YAP/TAZ and reinforce glycolytic programmes. The clinical record underscores the translational gap: PANTHER-IPF did not establish a benefit of N-acetylcysteine monotherapy; the phase II PRAISE trial reported a 60.3% relative reduction in the decline of predicted forced vital capacity at week 48 with pamrevlumab, but the subsequent phase III ZEPHYRUS-1 trial did not meet its primary endpoint of absolute FVC change at week 48 and showed no significant benefit on secondary outcomes (9). The phase III STARSCAPE trial of zinpentraxin alfa (PRM-151) was also terminated early for futility.

## Shared mediators of fibrosis

Three contributions test whether mechanisms are interchangeable between organs. Hua et al. interrogate four bulk and single-nucleus datasets from failing hearts and fibrotic kidneys and converge on PHLDA1 and IL-10, with areas under the curve of 0.931 and 0.941 in the merged discovery data and 0.872 and 0.829 on independent validation. PHLDA1 is highest in cardiac M1 macrophages and in injured proximal tubular epithelial cells, its deletion suppresses NF-κB signalling and lowers alpha-smooth muscle actin and COL1A1 in co-cultured PDGFRβ positive cells, which is induced by IL-1β and suppressed by TGF-β. That opposition recapitulates at the level of a single gene the inflammatory and matrix-producing fibroblast programs described above. However, Jin et al. report that their autophagy-related keloid markers ADRB2 and PLK2 reached areas under the curve of 0.991 and 0.974 in discovery and 0.778 and 0.889 in external validation, and the authors state plainly that the first set is optimistic and that their immune inferences reflect gene-set enrichment rather than measured cell abundance. Li et al. provide the field-level view, mapping 1,312 Web of Science records on macrophages in liver fibrosis published between 2013 and 2023 across 59 countries, with macrophage polarisation, non-alcoholic fatty liver disease and the NLRP3 inflammasome emerging as current frontier terms and institutional collaboration remaining largely intranational.

## EMT in less-studied organ systems

Three reviews in the collection extend the inflammation to fibrosis sequence into new and unexplored territories. Zhou et al. describe a self-amplifying EMT to fibrosis loop in plasma cell mastitis, in which ductal ectasia generates damage-associated molecular patterns. Pattern recognition receptors, including TLR4 and NLRP3, activate NF-κB, and IL-6/JAK/STAT3 sustains both plasma cell survival through Bcl-2 and Snail-driven EMT in ductal epithelium with postoperative recurrence of 43% being the clinical cost. Chen et al. review proliferative vitreoretinopathy, where a substantial fraction of α-smooth muscle actin-positive myofibroblasts has been attributed to keratin-positive retinal pigment epithelium and a further contribution comes from macrophage-to-myofibroblast transition, and where 27 randomised trials in 3,375 patients have not improved reattachment or visual acuity. The authors suggest that limited trial success may reflect, at least in part, patient selection, intervention timing and endpoint choice; this highlights the need to examine trial design before concluding that a biologically plausible target lacks therapeutic value. Wang et al. examine pelvic floor connective tissue, where estrogen acting through ERα, ERβ and GPER governs the matrix metalloproteinase to tissue inhibitor balance, macrophage polarisation and mechanotransduction through YAP and TAZ, and where local therapy improves tissue quality without preventing recurrence at three years.

## Therapeutic strategy and the translational gap

The therapeutic implications of various stages are highlighted in several articles in the collection. Xiao et al. review mesenchymal stromal cell-derived extracellular vesicles (MSC-EVs) in pulmonary fibrosis, discussing immunological mechanisms including M1-to-M2 reprogramming, Treg induction, NOD1/NF-κB/NLRP3 and Smad2 suppression, and mitochondrial transfer to alveolar macrophages. They also describe a randomised, single-blind, placebo-controlled phase I trial of nebulised human umbilical-cord mesenchymal stromal cell-derived extracellular vesicles in 24 patients that reported functional improvement without severe adverse events, while emphasising that isolation, characterisation and biodistribution remain unstandardised. Hong et al. combine network pharmacology, docking and validation in carbon tetrachloride-treated mice and in RAW264.7 macrophages to attribute the antifibrotic action of Pien Tze Huang to EGFR/JAK1/STAT3 blockade with suppression of CD68, IRF5 and IL-12A while stating that computational affinity does not establish target engagement. Shu et al. demonstrate natural products acting on NF-κB, MAPK, TGF-β/Smad, JAK2/STAT3 and the NLRP3 inflammasome in myocardial fibrosis, while mentioning their own limitations regarding hepatotoxicity, low oral bioavailability and supraphysiological animal doses.

## What the collection establishes, and what it leaves open

Taken together, these 20 articles support a model in which inflammatory fibroblast states and immune–fibroblast interactions are biologically active and potentially therapeutically addressable components of fibrogenesis, although their temporal and causal relationships remain tissue- and disease-stage dependent. Across organs, the findings are consistent with a dynamic feedback model in which immune cues, fibroblast plasticity, metabolic and epigenetic regulation, and matrix mechanics influence whether injury resolves or progresses to self-sustaining fibrosis; shared pathways should not be assumed to have equivalent functions or therapeutic consequences across tissues. More recent lineage-tracing and single-cell analyses also implicate RUNX2 in the transition from alveolar fibroblasts to pathological fibroblasts in experimental pulmonary fibrosis (10). The claim is strongest where a single intervention separates the two outcomes, as when PHLDA1 deletion lowers NF-κB signalling and matrix gene expression in neighbouring cells (Hua et al.), when IKZF1 inhibition reduces peritoneal cytokines and pulmonary collagen together (Liu et al.), and when interferon receptor blockade limits vascular remodelling in the neonatal lung (Zheng et al.). It is weakest where one molecule acts in opposite directions depending on context, as with IL-17 blockade, which can benefit some inflammatory skin conditions but may be associated with intestinal adverse effects (Cai et al.), and with the opposing effects of IL-1β and TGF-β on PHLDA1 expression (Hua et al.).

### Five priorities follow

The inflammatory fibroblast needs to be settled as an obligatory intermediate or a persistent partner, which calls for lineage tracing rather than frequency measurement. Developmental and disease stage need to enter trial design, since the neonatal and adult immune programs differ and intervention timing already accounts for negative trials in the eye. Candidate biomarkers should be evaluated in independent external cohorts, and performance estimates obtained from discovery datasets should be identified as potentially optimistic. Tissue-specific immune–stromal circuits should be defined experimentally rather than assuming that conserved pathways have equivalent functions or therapeutic effects across organs. Finally, selective inhibitors should be evaluated for their ability to preserve protective immune functions in other tissues before the IL-17 and TGF-β axes are targeted in patients.
